# Formulation-dependent colloidal properties of redispersible lycopene nanoparticles: storage stability and enhanced *in vitro* bioactivity

**DOI:** 10.1039/d6ra08147a

**Published:** 2026-10-19

**Authors:** Ho Thi Oanh, Hong Tham Nguyen, Nguyen Phu Duc, Nhung Hac Thi, Dinh Dung Luong, Mai Ha Hoang

**Affiliations:** a Institute of Chemistry, Vietnam Academy of Science and Technology 18 Hoang Quoc Viet, Nghia Do Ward Hanoi 10000 Vietnam hoangmaiha@ich.vast.vn; b Graduate University of Science and Technology, Vietnam Academy of Science and Technology 18 Hoang Quoc Viet, Nghia Do Ward Hanoi 10000 Vietnam

## Abstract

Lycopene is a bioactive carotenoid whose application is limited by poor aqueous dispersibility and susceptibility to degradation. Redispersible lycopene nanoparticles were prepared by solvent displacement–evaporation followed by freeze-drying, and interfacial composition and nominal lycopene fraction were evaluated sequentially. Among the tested interfacial compositions, a 2 : 1 Tween 80/phosphatidylcholine mass ratio produced the smallest particles and lowest dispersity. At this ratio, the formulation containing 12 wt% nominal lycopene was selected for its balance of lycopene content, particle size, and dispersity. The measured lycopene content of the freeze-dried formulation was 11.63 wt%; after aqueous redispersion, the particles exhibited a mean hydrodynamic diameter of 133.25 ± 1.46 nm, a polydispersity index of 0.173 ± 0.016, and a zeta potential of −38.3 ± 1.9 mV. TEM showed predominantly near-spherical particles. FTIR spectra were consistent with physical incorporation in the matrix, while XRD showed marked attenuation of crystalline lycopene reflections. The redispersed formulation maintained its colloidal characteristics for 7 days at 4 °C, and the freeze-dried formulation retained 91.9% of its initial lycopene content after 180 days at −20 °C. At matched lycopene doses, the formulation showed higher apparent DPPH radical-scavenging activity and greater protection against H_2_O_2_-induced loss of HepG2 cell viability than free lycopene. It also inhibited oleic acid-induced intracellular lipid accumulation by 89.1% at the highest dose *versus* 68.0% for free lycopene, while cell viability remained above 90%. Overall, sequential evaluation of interfacial composition and nominal lycopene fraction yielded a redispersible freeze-dried formulation combining controlled colloidal properties, storage stability, and improved *in vitro* functional performance.

## Abstract 2

A redispersible lycopene freeze-dried powder combines a 133 nm hydrodynamic diameter, 11.63 wt% lycopene content, high storage retention, and enhanced *in vitro* functional performance.

## Graphical abstract

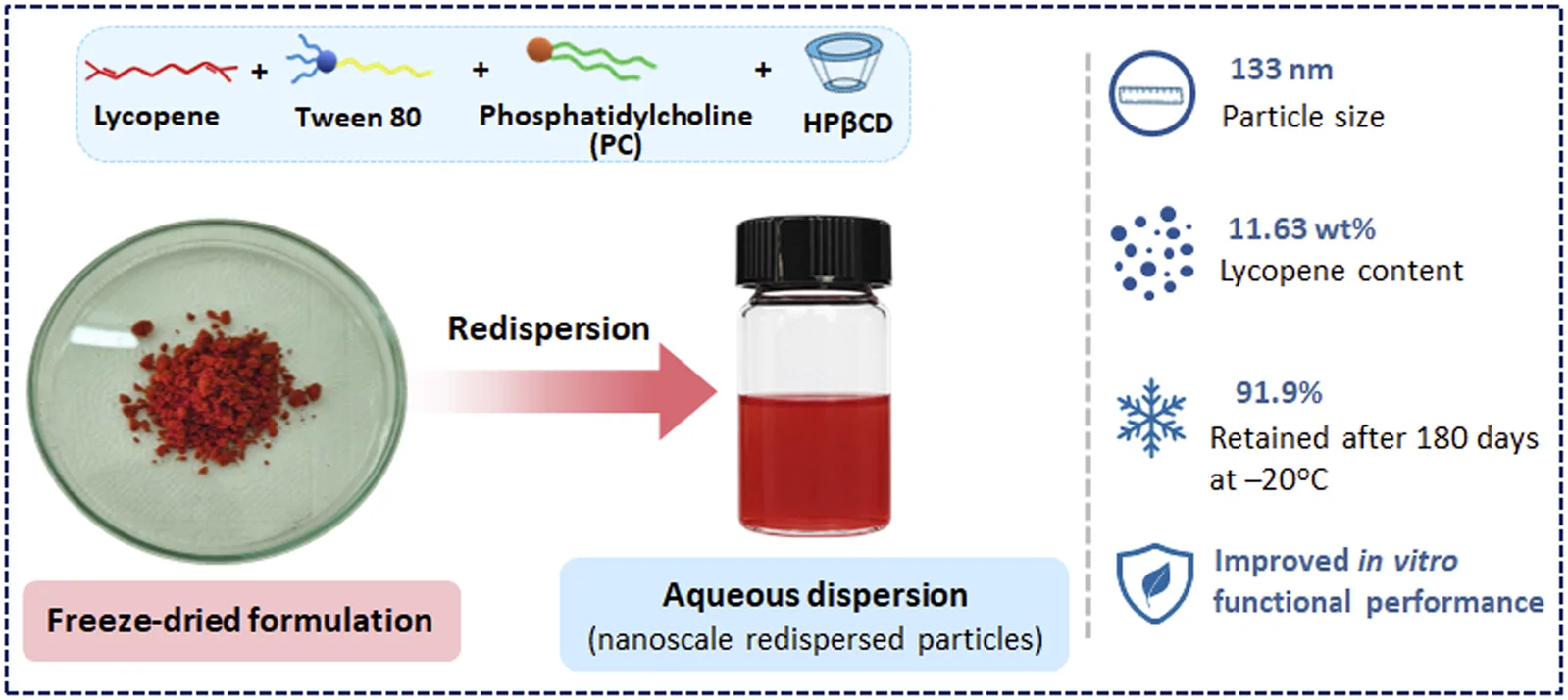

## 1 Introduction

Lycopene is an acyclic carotenoid widely distributed in red fruits and vegetables, notably tomatoes and gac fruit.^1,2^ Its extended conjugated polyene structure enables efficient singlet oxygen quenching and free-radical scavenging and contributes to its reported biological activities.^3–5^ Dietary lycopene and lycopene-rich foods have been associated with favorable effects on oxidative stress and cardiometabolic health, although the magnitude of these effects varies with dose, food matrix, formulation, and baseline physiological status.^6,7^ The broader application of lycopene in food and nutraceutical products, however, remains constrained by its very low aqueous solubility and susceptibility to oxidation, degradation, and geometrical isomerization upon exposure to light, oxygen, heat, and acidic conditions.^8–10^ Effective delivery systems must therefore improve aqueous dispersibility while protecting lycopene during processing and storage.

A variety of colloidal and nanoscale delivery systems have been investigated to address these limitations, including lipid-based nanocarriers, polymeric nanoparticles, polyelectrolyte complexes, vesicular carriers, and aqueous nanodispersions.^11–14^ These approaches can improve lycopene dispersibility, stability, bioaccessibility, and functional performance. However, carrier composition, interfacial stabilizers, component ratios, lycopene loading, and processing conditions often vary simultaneously among reported systems, making the contribution of individual factors difficult to distinguish.^11,15–17^ Controlled comparisons within a fixed platform are therefore needed to establish formulation–property relationships rather than merely identify systems with favorable initial characteristics.

Interfacial composition is a particularly important determinant of particle formation and colloidal behavior.^15,17^ Tween 80, a nonionic surfactant, can provide steric stabilization, whereas phosphatidylcholine (PC), a zwitterionic phospholipid and one of the principal phospholipid constituents of lecithin, can contribute to interfacial organization.^15–17^ In lycopene nanodispersions, Tween 80 has been reported to produce smaller particles than several alternative emulsifiers, whereas lecithin-based systems can exhibit relatively narrow particle-size distributions and favorable physical stability.^15^ Phospholipid-based vesicular carriers and Tween 80/lecithin-containing lipid nanoparticles have also been reported for lycopene.^16,17^ Nevertheless, systematic comparisons of Tween 80, phosphatidylcholine, and their combination under otherwise fixed formulation conditions remain limited.

Hydroxypropyl-β-cyclodextrin (HPβCD) is a highly water-soluble β-cyclodextrin derivative widely used as a solubilizing and stabilizing excipient for poorly water-soluble compounds.^18^ Lycopene–cyclodextrin systems have been reported to improve aqueous dispersibility and dissolution behavior,^19–22^ lycopene stability,^19,21^ and DPPH radical-scavenging activity.^21,22^ Experimental characterization and molecular dynamics simulations of an HPβCD/PEG 6000 ternary system further supported association of lycopene with HPβCD-containing aggregates.^20^ However, how interfacial composition governs lycopene content and colloidal behavior within a controlled HPβCD-assisted nanoparticle platform, and how increasing lycopene loading affects particle-size control and redispersibility, remain insufficiently defined.

These questions are particularly relevant to freeze-dried formulations intended for subsequent redispersion. Freezing and dehydration can promote particle aggregation and impair recovery of the original colloidal state.^23,24^ Moreover, recovery of colloidal properties does not necessarily ensure preservation of lycopene during prolonged storage. An effective dry formulation must therefore balance lycopene content, redispersibility, and storage stability. Comparison with free lycopene at matched doses is also important for distinguishing formulation-enabled effects from differences attributable simply to lycopene dose.

Accordingly, this study investigated an HPβCD-assisted lycopene nanoparticle platform using a sequential, controlled formulation strategy. Tween 80, l-α-phosphatidylcholine, and their combination were first compared at fixed lycopene and HPβCD contents and a fixed total amount of interfacial stabilizer to determine the influence of interfacial composition on the lycopene content of the recovered powder and its colloidal properties. The nominal lycopene loading was then systematically varied within the selected interfacial system to characterize loading-dependent changes in particle size, dispersity, zeta potential, and redispersibility and to identify a composition balancing lycopene content with controlled colloidal properties. The selected freeze-dried powder was subsequently characterized by transmission electron microscopy (TEM), Fourier transform infrared (FTIR) spectroscopy, and X-ray diffraction (XRD) and evaluated for colloidal stability after redispersion and lycopene retention during storage. Finally, its *in vitro* functional performance was compared with that of free lycopene at matched lycopene doses using DPPH radical scavenging, protection against H_2_O_2_-induced loss of HepG2 cell viability, and attenuation of oleic acid-induced intracellular lipid accumulation. This design enables formulation–property–performance relationships to be examined within a single redispersible lycopene nanoparticle platform.

## 2 Experimental

### 2.1. Materials

Lycopene (LYC, purity 98%) was extracted from gac (*Momordica cochinchinensis* Spreng.) fruit according to our previously reported procedure;^2^ its characterization is provided in Fig. S1. Tween 80 (T80), l-α-phosphatidylcholine (PC), and hydroxypropyl-β-cyclodextrin (HPβCD, ≥98%) were purchased from Sigma-Aldrich (USA). 2,2-Diphenyl-1-picrylhydrazyl (DPPH, 98%), oleic acid (OA), and lovastatin were obtained from AK Scientific (USA). High-glucose Dulbecco's modified Eagle's medium (DMEM) and 3-(4,5-dimethylthiazol-2-yl)-2,5-diphenyltetrazolium bromide (MTT) were purchased from Thermo Fisher Scientific (USA). Analytical-grade tetrahydrofuran (THF), dimethyl sulfoxide (DMSO), ethanol, and methanol were obtained from Sigma-Aldrich (USA). All chemicals and reagents were used as received without further purification.

### 2.2. Fabrication of lycopene nanoparticles

Lycopene nanoparticles were prepared by solvent displacement–evaporation followed by homogenization and freeze-drying. Six formulations (NL-1–NL-6), each with a nominal total solids mass of 200 mg, were prepared as summarized in Table 1. For the initial controlled comparison of interfacial stabilizer compositions, NL-1–NL-3 contained a nominal lycopene fraction of 5 wt% and differed only in the identity and ratio of the interfacial stabilizers: Tween 80 alone (30 mg), PC alone (30 mg), or Tween 80/PC (20/10 mg; 2 : 1, w/w), respectively. The Tween 80/PC composition used in NL-3 was subsequently retained in NL-4–NL-6, in which the nominal lycopene fraction was increased to 10, 12, and 15 wt%, respectively, while the amount of HPβCD was adjusted to maintain a constant nominal total solids mass. For each formulation, lycopene and the specified interfacial stabilizer(s) were dissolved in 5 mL of THF under magnetic stirring at 600 rpm for 30 min. HPβCD was separately dissolved in 50 mL of distilled water, and the organic phase was added dropwise to the aqueous phase under continuous stirring. The resulting dispersion was subsequently stirred at 650 rpm for 3 h to reduce the THF content by evaporation while being protected from light with aluminum foil. The dispersion was then homogenized at 5000 rpm for 15 min, prefrozen, and freeze-dried using a LABCONCO freeze-drying system (USA) to obtain a redispersible freeze-dried lycopene formulation. The overall preparation and redispersion procedure is illustrated schematically in Fig. 1.

**Table 1:** Composition of the lycopene nanoparticle formulations

Formulation	LYC (mg)	T80 (mg)	PC (mg)	HPβCD (mg)	Nominal LYC loading (wt%)
NL-1	10	30	—	160	5
NL-2	10	—	30	160	5
NL-3	10	20	10	160	5
NL-4	20	20	10	150	10
NL-5	24	20	10	146	12
NL-6	30	20	10	140	15

**Fig. 1:**
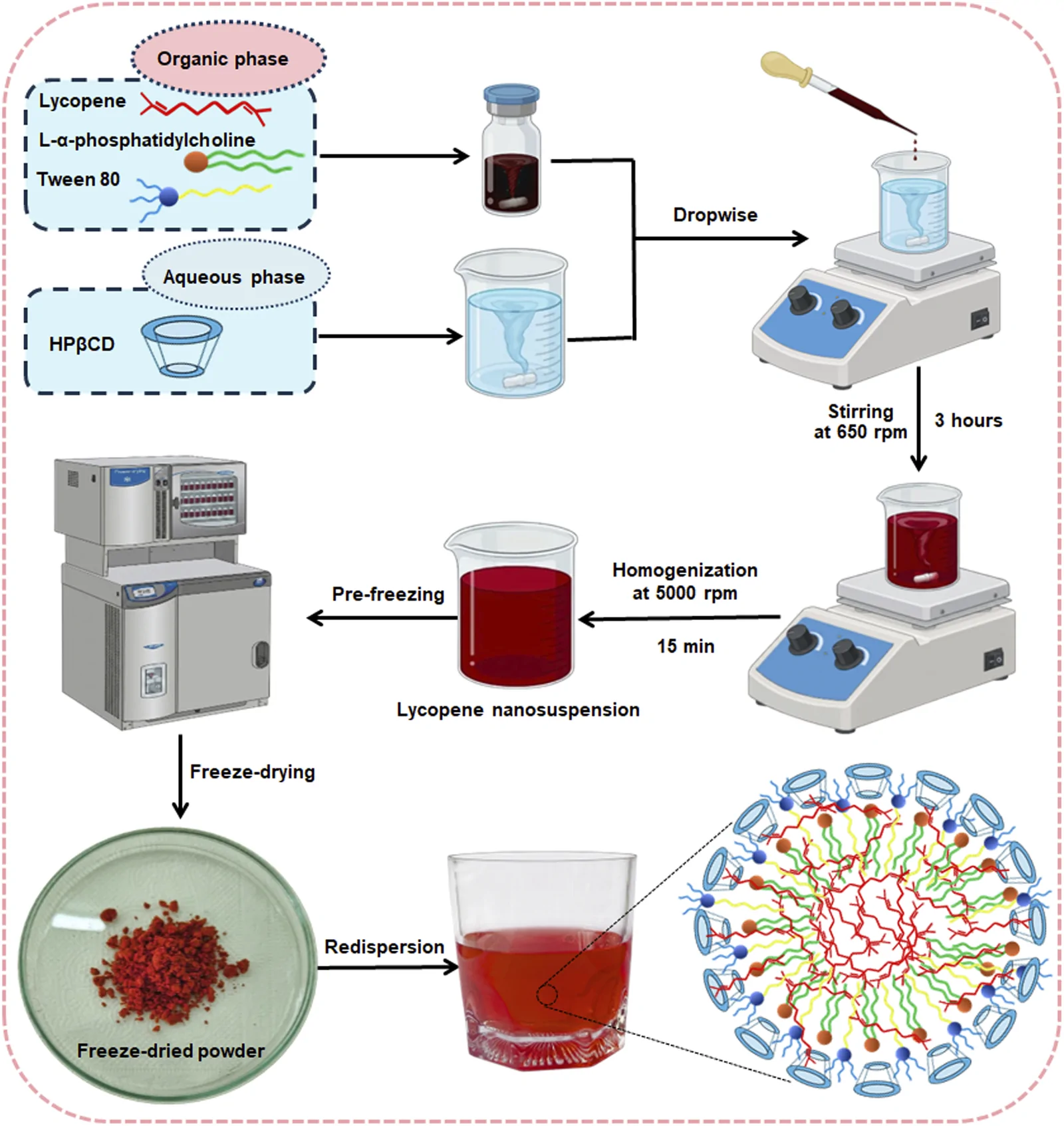
Schematic illustration of the preparation and aqueous redispersion of freeze-dried lycopene formulations. The post-redispersion particle representation is conceptual and does not imply a defined internal architecture.

### 2.3. Determination of lycopene content

An accurately weighed amount of freeze-dried formulation was dispersed in THF and sonicated for 1 min. Lycopene was quantified by UV-visible spectrophotometry at 478 nm using an Optima SP-3000 Nano spectrophotometer (Japan). Concentrations were determined from a lycopene calibration curve over 0.02–0.10 mg mL^−1^ (*R*^2^ = 0.999). Lycopene content was calculated as:

1 Lycopene content (wt%) = (*C* × *V* × DF/*m*) × 100

where *C* is the lycopene concentration, *V* is the extraction volume, DF is the dilution factor, and *m* is the sample mass.

### 2.4. Physicochemical and structural characterization

Mean hydrodynamic diameter and polydispersity index (PDI) were determined by dynamic light scattering (DLS), and zeta potential was measured by electrophoretic light scattering using a Litesizer 500 (Anton Paar, Austria). Freeze-dried formulations were redispersed in distilled water at 0.5 mg mL^−1^ and allowed to stand at room temperature for 2 h without sonication before analysis.

Particle morphology was examined by transmission electron microscopy (TEM) using a JEM-2100 microscope (JEOL Ltd, Tokyo, Japan) equipped with a LaB_6_ electron source and operated at 200 kV. A drop of each redispersion was deposited on a carbon-coated copper grid, and excess liquid was removed by blotting. The specimens were negatively stained with 1% (w/v) phosphotungstic acid for 10 s and air-dried before imaging.

Attenuated total reflectance Fourier transform infrared (ATR-FTIR) spectra were acquired using a Spectrum Two spectrometer equipped with a diamond ATR accessory (PerkinElmer Inc., Llantrisant, UK). Before each measurement, the crystal was cleaned with 70% (v/v) ethanol and dried, and a background spectrum was recorded. The freeze-dried formulations were analyzed directly as powders, with pressure applied to ensure contact with the crystal. Spectra were collected over 4000–500 cm^−1^ at a resolution of 4 cm^−1^ using 32 co-added scans.

XRD patterns were obtained using a D8-Advance diffractometer (Bruker, Germany) with Cu Kα radiation (*λ* = 1.5406 Å) operated at 40 kV and 30 mA over a 2*θ* range of 10–70°.

### 2.5. Stability of lycopene nanoparticles

#### 2.5.1. Colloidal stability after redispersion

Freeze-dried NL-5 powder (10 mg) was redispersed in 20 mL of distilled water (0.5 mg mL^−1^) by allowing the dispersion to stand at room temperature for 2 h without sonication. The redispersed samples were stored in sealed, light-protected glass vials at 4 or 37 °C for 7 days. Mean hydrodynamic diameter, PDI, and zeta potential were measured on days 0, 1, 3, 5, and 7 as described in Section 2.4.

#### 2.5.2. Storage stability of freeze-dried powder

Freeze-dried NL-5 was stored in tightly sealed amber glass vials at −20, 4, or 37 °C for 180 days. Samples were analyzed on days 0, 15, 30, 60, 90, 120, and 180. At each time point, an identical mass of powder was processed under the extraction and dilution conditions described in Section 2.3. Lycopene was quantified at 478 nm, and retention was calculated relative to the initial value:2
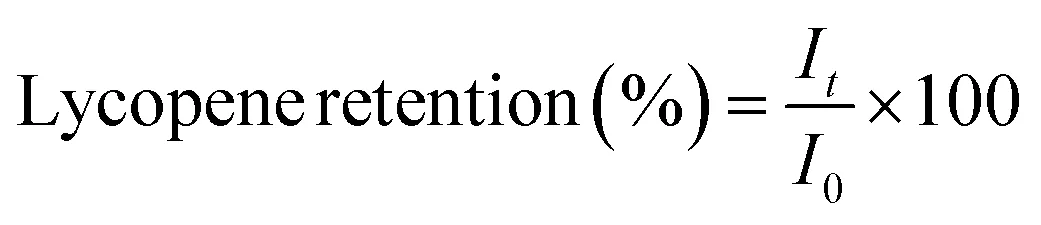
where *I*_0_ and *I*_*t*_ are the absorbance values at day 0 and after storage for time *t*, respectively.

### 2.6. DPPH radical-scavenging assay

DPPH radical-scavenging activity was determined according to Hien *et al.* with minor modifications.^25^ A 0.25 mM DPPH solution in methanol was freshly prepared. NL-5 was evaluated at formulation concentrations of 0.8, 4, 20, and 100 µg mL^−1^. Based on its measured lycopene content of 11.63 wt%, these concentrations corresponded to 0.093, 0.465, 2.326, and 11.63 µg per mL lycopene, respectively. Free lycopene was tested at the corresponding lycopene-equivalent concentrations. The blank HPβCD/Tween 80/PC formulation without lycopene (BL-NL-5) was tested at the corresponding excipient-equivalent concentrations. Each test sample was mixed with DPPH solution in a 96-well plate and incubated in the dark at room temperature for 30 min. Absorbance was measured at 517 nm. Corresponding sample blanks without DPPH were used for background correction, and ascorbic acid served as the positive control. DPPH radical-scavenging activity was calculated as:3
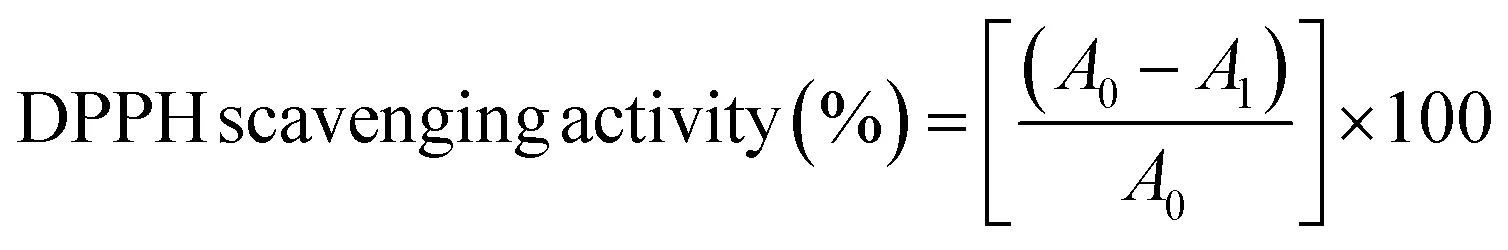
where *A*_0_ is the absorbance of the DPPH control and *A*_1_ is the blank-corrected absorbance of the test sample.

### 2.7. HepG2 cell culture

HepG2 cells (ATCC HB-8065) were cultured in high-glucose DMEM supplemented with 10% FBS, 100 U per mL penicillin, and 100 µg per mL streptomycin at 37 °C in a humidified 5% CO_2_ atmosphere. Cells were subcultured at approximately 80% confluence using trypsin–EDTA.

### 2.8. Cell viability and protection against H_2_O_2_-induced oxidative injury

Free lycopene was dissolved in DMSO and freshly diluted with culture medium immediately before cell treatment. The corresponding vehicle-control wells received an equivalent amount of DMSO to that introduced with the free-lycopene treatment. NL-5 and BL-NL-5 were separately redispersed in distilled water and subsequently diluted with culture medium to the required concentrations. BL-NL-5 served as the carrier-matrix control. HepG2 cells were seeded in 96-well plates at 3 × 10^4^ cells per well and allowed to attach overnight. For the oxidative-stress experiment, cells were pretreated for 24 h with free lycopene (1.163 µg mL^−1^), NL-5 formulation (10 µg mL^−1^; equivalent to 1.163 µg per mL lycopene), BL-NL-5 (8.837 µg mL^−1^; excipient-equivalent to 10 µg per mL NL-5), or ascorbic acid (20 µg mL^−1^). Cells were subsequently exposed to 0.5 mM H_2_O_2_ for 1 h. Untreated cells and cells exposed to H_2_O_2_ without sample pretreatment served as the normal and oxidative-stress controls, respectively. A parallel group treated with BL-NL-5 without H_2_O_2_ exposure was included to assess carrier-associated cytotoxicity.

Cell viability was determined using the MTT assay. After treatment, 50 µL of MTT solution (1 mg mL^−1^) was added to each well, followed by incubation for 4 h. The resulting formazan crystals were dissolved in 100 µL of DMSO, and absorbance was measured at 540 nm. Cell viability was expressed relative to the untreated control.

### 2.9. Intracellular lipid accumulation by Oil Red O staining

HepG2 cells were seeded in 96-well plates at 3 × 10^4^ cells per well and treated for 24 h with 0.5 mM OA in the presence of NL-5 at formulation concentrations of 0.8, 4, 20, or 100 µg mL^−1^, equivalent to 0.093, 0.465, 2.326, and 11.63 µg per mL lycopene, respectively. Free lycopene and BL-NL-5 were tested at the corresponding lycopene- and excipient-equivalent concentrations, respectively. OA-treated cells receiving 0.5% DMSO served as the OA vehicle control, cells receiving vehicle without OA served as the normal control, and lovastatin (10 µM) was used as the positive control.

After treatment, cells were washed with PBS, fixed with formalin for 30 min, and stained with 0.3% Oil Red O for 30 min. Excess stain was removed, and intracellular Oil Red O was eluted with 200 µL of 2-propanol for 10 min and quantified by measuring absorbance at 540 nm. Inhibition of intracellular lipid accumulation was calculated as:4
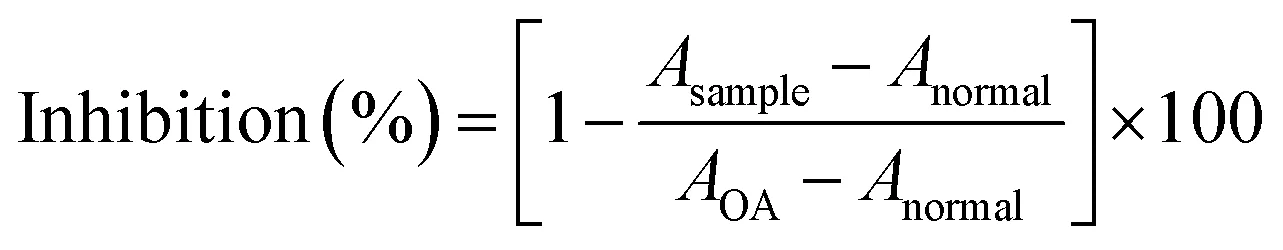
where *A*_sample_, *A*_OA_, and *A*_normal_ are the absorbance values of the sample-treated, OA-control, and normal-control groups, respectively.

For the cell-viability assessment associated with the OA model, parallel cultures were treated with free lycopene or NL-5 at the specified concentrations and evaluated by the MTT procedure described in Section 2.8. Viability was expressed relative to the OA-treated vehicle control.

### 2.10. Statistical analysis

Data are presented as mean ± standard deviation (SD) (*n* = 3). Differences among groups were analyzed by one-way analysis of variance (ANOVA) followed by Fisher's least significant difference test. Differences were considered statistically significant at *p* < 0.05.

## 3 Results and discussion

### 3.1. Effect of interfacial composition on the physicochemical properties of lycopene nanoparticles

To evaluate the effect of interfacial composition, NL-1–NL-3 were prepared with fixed lycopene and HPβCD contents while maintaining a constant total amount of stabilizer (Table 1). NL-1 and NL-2 contained Tween 80 and phosphatidylcholine (PC) alone, respectively, whereas NL-3 contained Tween 80 and PC at a mass ratio of 2 : 1. All three formulations produced red freeze-dried powders that could be redispersed in water to form visually homogeneous dispersions without visible sedimentation at the time of observation (Fig. 2A).

**Fig. 2:**
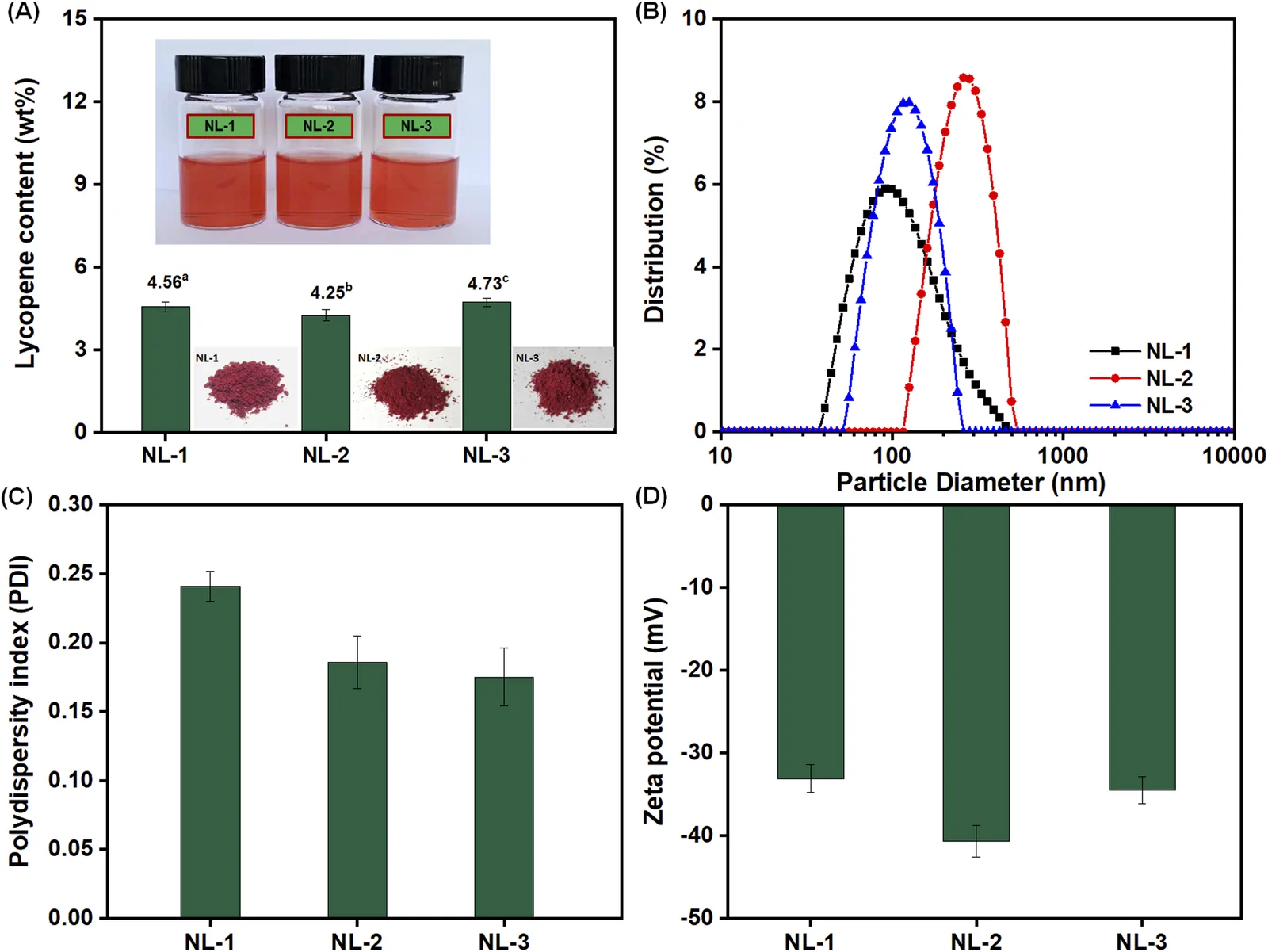
Effect of interfacial composition on the properties of the lycopene formulations. (A) Measured lycopene content of the recovered freeze-dried powders, with photographs of the corresponding powders and aqueous redispersions; (B) representative intensity-weighted particle-size distributions after redispersion; (C) polydispersity index (PDI); and (D) zeta potential of NL-1 (Tween 80), NL-2 (PC), and NL-3 (Tween 80/PC, 2 : 1, w/w). Quantitative data in (A), (C), and (D) are presented as mean ± SD (*n* = 3).

At a common nominal lycopene fraction of 5 wt%, the measured lycopene content differed significantly among formulations (*p* < 0.05; Fig. 2A). NL-3 showed the highest value (4.73 wt%), followed by NL-1 (4.56 wt%) and NL-2 (4.25 wt%). These values reflect the lycopene content of the recovered freeze-dried solids rather than a measure of process recovery or encapsulation efficiency.

After aqueous redispersion, the mean hydrodynamic diameters of NL-1, NL-2, and NL-3 were 127.01 ± 1.85, 260.70 ± 1.67, and 117.88 ± 1.82 nm, respectively (Fig. 2B), with corresponding PDI values of 0.241 ± 0.011, 0.186 ± 0.019, and 0.175 ± 0.021 (Fig. 2C). NL-2 exhibited a mean diameter more than twice that of NL-3, whereas NL-1 had the highest mean PDI despite its intermediate mean diameter. Stabilizer composition therefore affected characteristic size and dispersity differently, consistent with previous observations for lycopene nanodispersions.^15^

The larger diameter of NL-2 may reflect PC-rich assemblies, including vesicle-like structures,^16^ or association between dispersed particles. In the mixed Tween 80/PC system, the smaller diameter and lower mean PDI observed for NL-3 are consistent with previously reported effects of emulsifier composition on lycopene nanodispersion properties^15^ and with reports that combined Tween 80/lecithin systems can yield smaller particles and lower PDI, with Tween 80 contributing steric stabilization.^17^

The zeta potentials of NL-1, NL-2, and NL-3 were −33.1 ± 1.7, −40.7 ± 1.9, and −34.5 ± 1.6 mV, respectively (Fig. 2D). Although Tween 80 is nonionic and PC is zwitterionic, colloidal interfaces containing these amphiphiles can exhibit negative zeta potentials through preferential adsorption of anions, including hydroxide ions, and other specific ion–interface interactions in the aqueous medium.^26^ Such interfacial processes provide a plausible origin for the negative values observed here, although their individual contributions were not experimentally resolved. NL-2 exhibited the greatest zeta-potential magnitude but also the largest mean hydrodynamic diameter, indicating that zeta-potential magnitude alone did not account for the observed size differences.

Among the compositions examined, NL-3 combined the highest measured lycopene content with the smallest hydrodynamic diameter and lowest mean PDI. Accordingly, the Tween 80/PC mass ratio of 2 : 1 was retained for the subsequent loading study.

### 3.2. Physicochemical changes across formulations with increasing nominal lycopene fraction

At the selected Tween 80/PC mass ratio of 2 : 1, the nominal lycopene fraction was increased from 5 wt% in NL-3 to 10, 12, and 15 wt% in NL-4, NL-5, and NL-6, respectively (Table 1). The absolute amounts of Tween 80 and PC remained constant, whereas HPβCD was progressively reduced to maintain a constant nominal total solids mass. The measured lycopene content consequently rose from 4.73 wt% in NL-3 to 9.52, 11.63, and 14.24 wt% in NL-4, NL-5, and NL-6, respectively (Fig. 3A), confirming that the compositional increase was reflected in the recovered freeze-dried formulations.

**Fig. 3:**
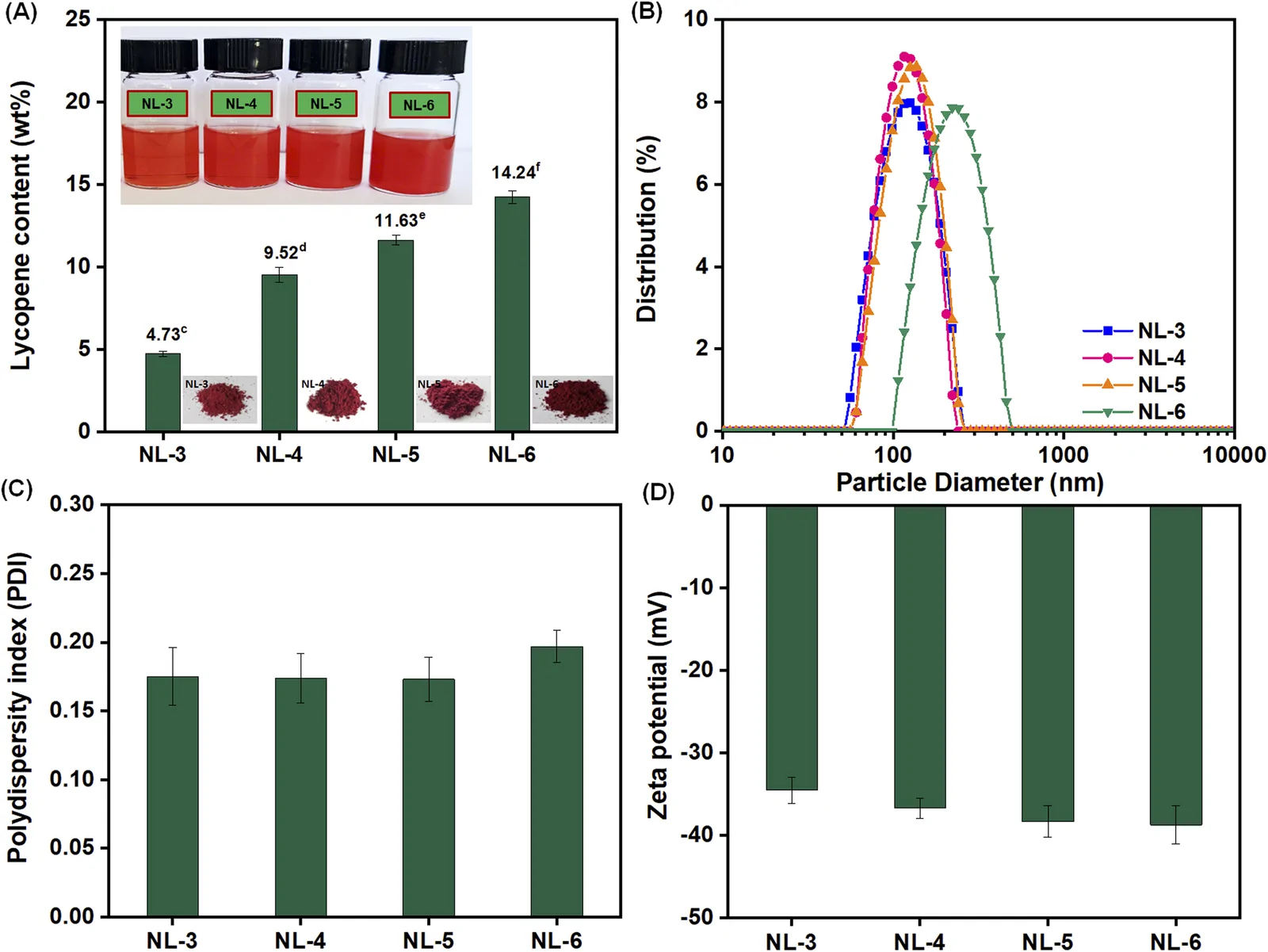
Effect of nominal lycopene fraction on the lycopene content of the freeze-dried formulations and their colloidal properties after aqueous redispersion. (A) Measured lycopene content, with photographs of the corresponding powders and aqueous redispersions; (B) representative intensity-weighted hydrodynamic size distributions; (C) polydispersity index (PDI); and (D) zeta potential. NL-3, NL-4, NL-5, and NL-6 contained 5, 10, 12, and 15 wt% nominal lycopene, respectively. Data in (A), (C) and (D) are presented as mean ± SD (*n* = 3).

Between 5 and 12 wt% nominal lycopene, the increase in measured content was accompanied by only limited particle enlargement. Mean hydrodynamic diameter increased from 117.88 ± 1.82 nm for NL-3 to 123.83 ± 2.14 nm for NL-4 and 133.25 ± 1.46 nm for NL-5 (Fig. 3B), whereas the corresponding PDI values remained within a narrow range of 0.173–0.175 (Fig. 3C). Thus, the increase in measured lycopene content up to NL-5 occurred without appreciable broadening of the hydrodynamic size distribution.

A more pronounced size change emerged at 15 wt% nominal lycopene. NL-6 exhibited a mean hydrodynamic diameter of 234.54 ± 4.32 nm, together with a shift of the representative intensity-weighted distribution toward larger diameters (Fig. 3B). Its PDI increased only moderately to 0.197 ± 0.012, indicating that the increase in mean size was not accompanied by marked broadening of the distribution. Zeta potential varied comparatively little across the series, from −34.5 ± 1.6 mV for NL-3 to −36.7 ± 1.2, −38.3 ± 1.9, and −38.7 ± 2.3 mV for NL-4, NL-5, and NL-6, respectively (Fig. 3D). The enlargement of NL-6 therefore occurred without a corresponding decrease in the magnitude of its negative zeta potential.

The compositional progression from NL-3 to NL-6 simultaneously decreased the HPβCD-to-lycopene and Tween 80/PC-to-lycopene mass ratios because lycopene replaced HPβCD while the absolute amounts of the interfacial stabilizers were held constant. The larger hydrodynamic size of NL-6 may consequently reflect the lower relative availability of carrier and interfacial stabilizers per unit of lycopene. These compositional effects cannot, however, be separated within the present design. The increase in diameter between 12 and 15 wt% nominal lycopene is therefore best interpreted as a formulation-associated change within the investigated series, rather than as evidence of an intrinsic critical lycopene-loading threshold.

Lycopene has previously been incorporated into liquid nanodispersions, vesicular carriers, and lipid-based nanoparticles, demonstrating that substantially different carrier architectures can achieve nanoscale dispersion of this highly hydrophobic carotenoid.^15–17^ The HPβCD-assisted system investigated here addresses a complementary formulation objective by coupling compositional optimization with a freeze-dried format intended for subsequent aqueous redispersion. This distinction is relevant because colloidal performance is evaluated after reconstitution of the dried formulation, rather than solely in the initially prepared dispersion.

Ma *et al.* reported lycopene-loaded nanostructured lipid carriers with a mean diameter of 126.48 ± 3.87 nm, a PDI of 0.188 ± 0.028, and a loading of 2.56 ± 0.01 wt%.^27^ Nazemiyeh *et al.* described solid lipid nanoparticles approximately 125 nm in diameter with a loading of 44.8 ± 0.46 mg g^−1^ (approximately 4.48 wt%).^28^ NL-5 contained 11.63 wt% total lycopene, measured per unit mass of recovered powder, and exhibited nanoscale dimensions and low dispersity after aqueous redispersion. Differences in the analytical basis of loading measurements, carrier architecture, and intended application limit direct comparisons of formulation performance. Within the present series, NL-5 provided a higher lycopene content than NL-3 and NL-4 without the marked particle enlargement observed for NL-6 and was therefore selected for subsequent storage-stability and *in vitro* functional evaluations.

### 3.3. Morphology of redispersed lycopene nanoparticles

TEM specimens prepared from aqueous redispersions of the freeze-dried formulations showed predominantly near-spherical particles for NL-3–NL-5, with relatively uniform morphology and limited local association (Fig. 4A–C). NL-6 exhibited more pronounced particle association and larger electron-dense features (Fig. 4D). This difference was qualitatively consistent with the increase in mean hydrodynamic diameter measured by DLS at the highest nominal lycopene fraction (Fig. 3B). The micrographs represent negatively stained, air-dried specimens, in which particle contours and local association may be influenced by staining and drying.

**Fig. 4:**
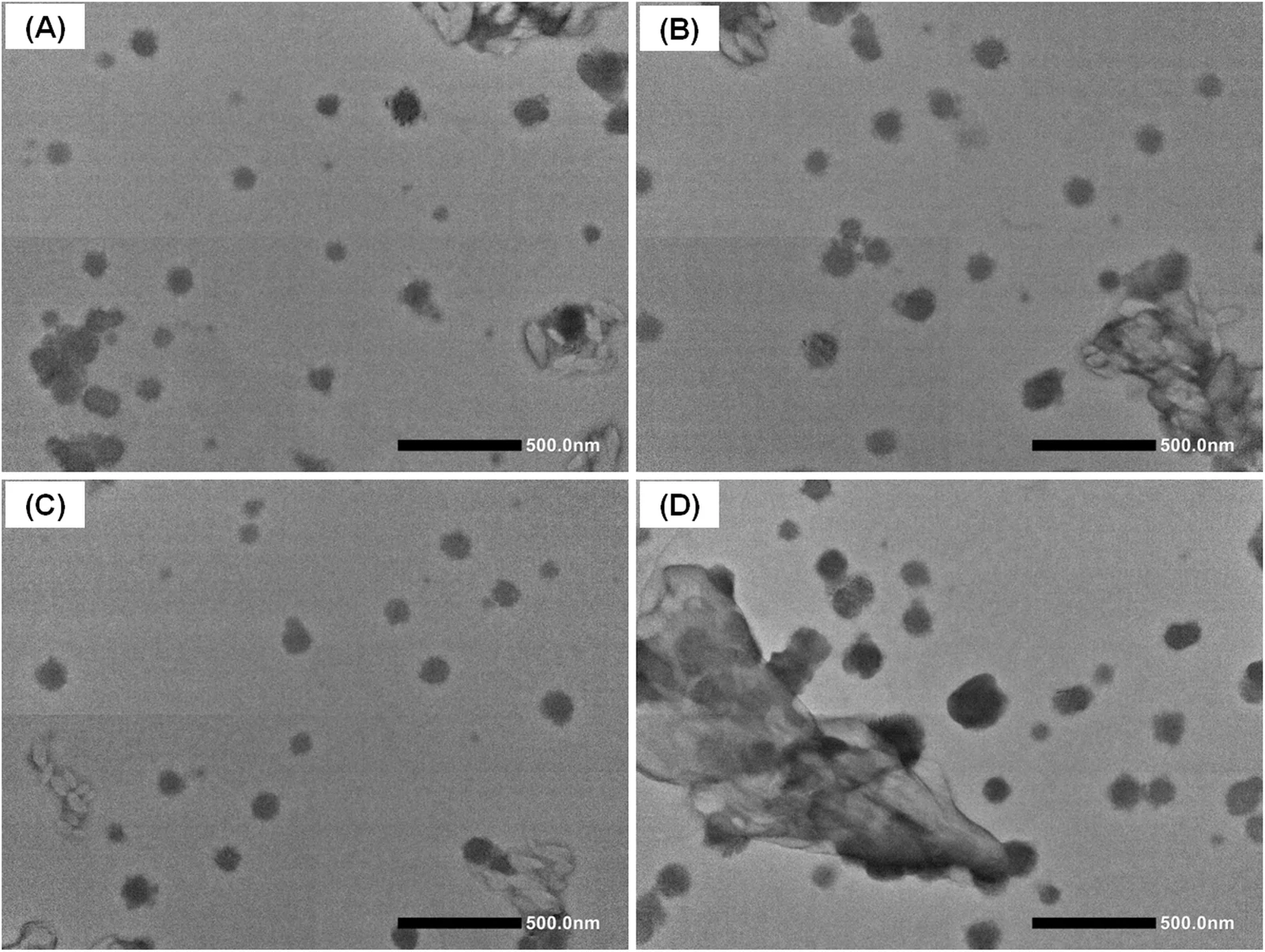
Representative TEM images of negatively stained lycopene nanoparticles after aqueous redispersion: (A) NL-3, 5 wt%; (B) NL-4, 10 wt%; (C) NL-5, 12 wt%; and (D) NL-6, 15 wt% nominal lycopene. All formulations were prepared using the selected Tween 80/phosphatidylcholine (PC) mass ratio of 2 : 1. Scale bars: 500 nm.

### 3.4. FTIR and XRD analyses

FTIR spectroscopy and XRD were used to characterize the molecular features and solid-state organization of NL-3–NL-6 across the investigated lycopene-loading range (Fig. 5).

**Fig. 5:**
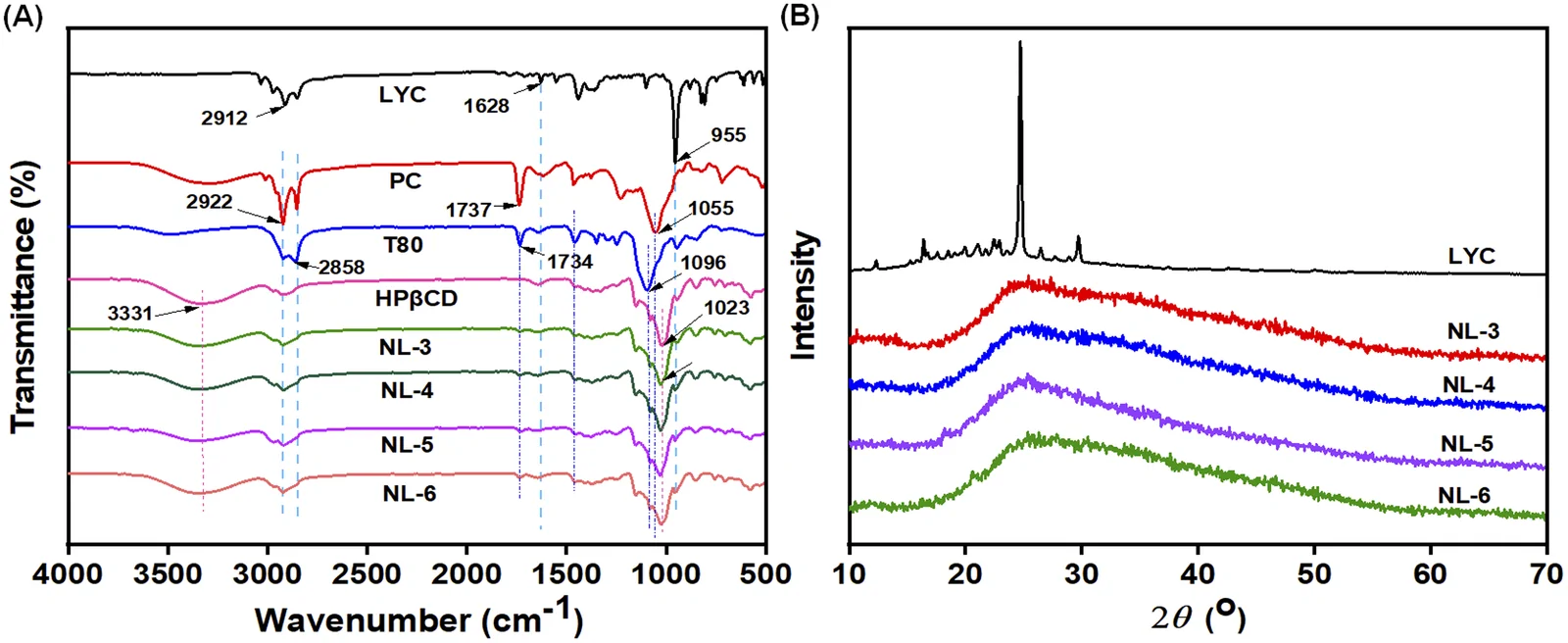
FTIR and XRD characterization of lycopene nanoparticles. (A) FTIR spectra of free lycopene, phosphatidylcholine, Tween 80, hydroxypropyl-β-cyclodextrin, and formulations NL-3–NL-6; and (B) XRD patterns of free lycopene and NL-3–NL-6.

Free lycopene exhibited characteristic FTIR bands at 2912, 1628, and 955 cm^−1^, assigned to C–H stretching, conjugated C=C stretching, and *trans*-polyene C–H out-of-plane deformation, respectively (Fig. 5A).^29^ PC showed characteristic bands at 2922 cm^−1^ in the aliphatic C–H stretching region, 1737 cm^−1^ corresponding to ester C=O stretching, and 1055 cm^−1^ in the phosphate/C–O stretching region.^30^ Tween 80 exhibited bands at 2858, 1734, and 1096 cm^−1^, attributable to aliphatic C–H, ester C=O, and ether C–O–C stretching, respectively.^31^ HPβCD was characterized by a broad O–H stretching band centered at 3331 cm^−1^ and a strong C–O/C–O–C absorption at 1023 cm^−1^.^32^

The spectra of NL-3–NL-6 were dominated by matrix-associated absorptions, including broad O–H stretching, C–H stretching in the 2858–2922 cm^−1^ region, ester C=O absorption in the 1734–1737 cm^−1^ region, and intense C–O/C–O–C bands in the 1000–1100 cm^−1^ region. The characteristic lycopene bands at 1628 and 955 cm^−1^ were markedly attenuated or obscured by matrix signals. No new absorption bands were observed in NL-3–NL-6, and their overall spectral profiles remained similar across the loading series. These observations are consistent with physical incorporation of lycopene within the formulation matrix and provide no spectroscopic evidence of new covalent bond formation. Increasing the nominal lycopene loading from 5 to 15 wt% did not substantially alter the overall FTIR profile of the freeze-dried formulations.

The XRD pattern of free lycopene displayed multiple sharp diffraction peaks in the 2*θ* range of 15–30°, including a prominent reflection at 2*θ* = 24.8°, consistent with its crystalline nature (Fig. 5B). In contrast, NL-3–NL-6 exhibited broad diffuse diffraction patterns with no distinct peaks attributable to crystalline lycopene. The suppression of the lycopene peaks indicates a substantial reduction in detectable crystalline order within the freeze-dried powders. Although these patterns are consistent with a largely disordered solid-state environment, a minor crystalline fraction below the detection limit of XRD cannot be excluded. Similar suppression of crystalline lycopene peaks following incorporation into nanoparticles has been reported previously.^33^

Notably, no distinct crystalline lycopene reflections emerged in NL-6 despite its higher lycopene content and substantially larger hydrodynamic size. Thus, the pronounced particle enlargement observed at 15 wt% nominal loading was not accompanied by detectable lycopene recrystallization. Collectively, the FTIR and XRD results indicate that increasing lycopene loading had a substantially greater influence on colloidal size than on the molecular spectral features or solid-state diffraction profile of the freeze-dried matrix.

### 3.5. Stability of lycopene nanoparticles

#### 3.5.1. Colloidal stability after redispersion

The colloidal stability of redispersed NL-5 was evaluated over 7 days at 4 and 37 °C by monitoring mean hydrodynamic diameter and zeta potential (Fig. 6), together with PDI (Fig. S2). At 4 °C, the mean hydrodynamic diameter changed only slightly from approximately 133 nm at day 0 to 137 nm at day 7 (Fig. 6A). The zeta potential remained relatively stable, changing from −38.3 to −36.8 mV (Fig. 6B), while only a modest increase in PDI was observed. These limited changes indicate that NL-5 largely retained its colloidal characteristics during 7 days of refrigerated storage after redispersion. At 37 °C, the mean hydrodynamic diameter increased after day 1, reaching approximately 142, 158, and 180 nm on days 3, 5, and 7, respectively (Fig. 6A). Over the same period, the zeta potential became progressively less negative and reached −28.7 mV by day 7 (Fig. 6B), whereas the PDI increased only moderately. The increase in hydrodynamic size without pronounced broadening of the size distribution may reflect progressive particle association or structural rearrangement rather than formation of a highly heterogeneous particle population. Temperature-dependent decreases in colloidal stability have also been reported for other lycopene-containing nanocarriers.^34^ These data indicate that refrigerated storage is preferable to 37 °C for maintaining the short-term colloidal characteristics of redispersed NL-5.

**Fig. 6:**
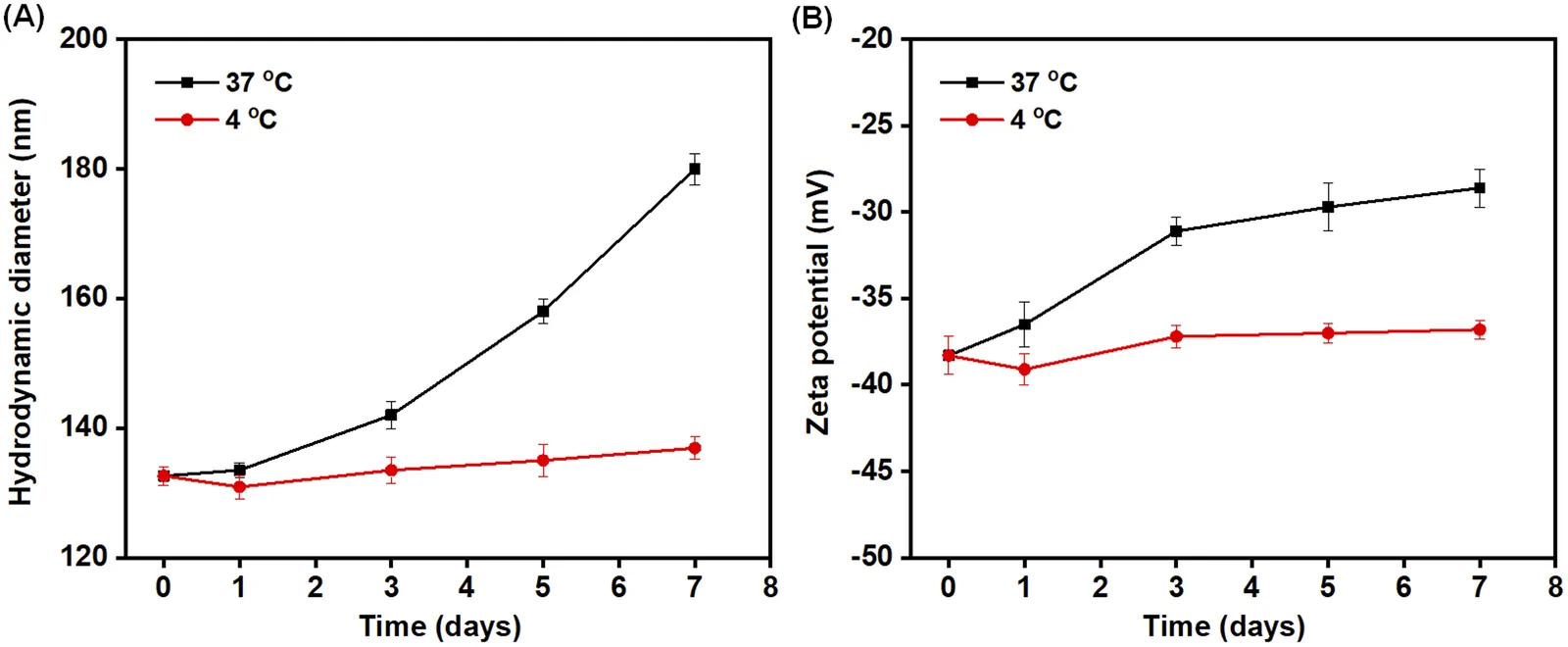
Colloidal stability of redispersed NL-5 during 7 days of storage at 4 and 37 °C. (A) Changes in mean hydrodynamic diameter and (B) zeta potential as a function of storage time. Data are presented as mean ± SD (*n* = 3).

#### 3.5.2. Storage stability of freeze-dried NL-5

The storage stability of freeze-dried NL-5 was evaluated at −20, 4, and 37 °C over 180 days. UV-Vis spectra recorded at each sampling time retained the characteristic lycopene absorption profile, including the analytical maximum near 478 nm, without a marked shift in peak position (Fig. 7A–C). Spectral intensity nevertheless decreased with storage time, with the greatest attenuation observed at 37 °C, an intermediate decrease at 4 °C, and comparatively minor changes at −20 °C.

**Fig. 7:**
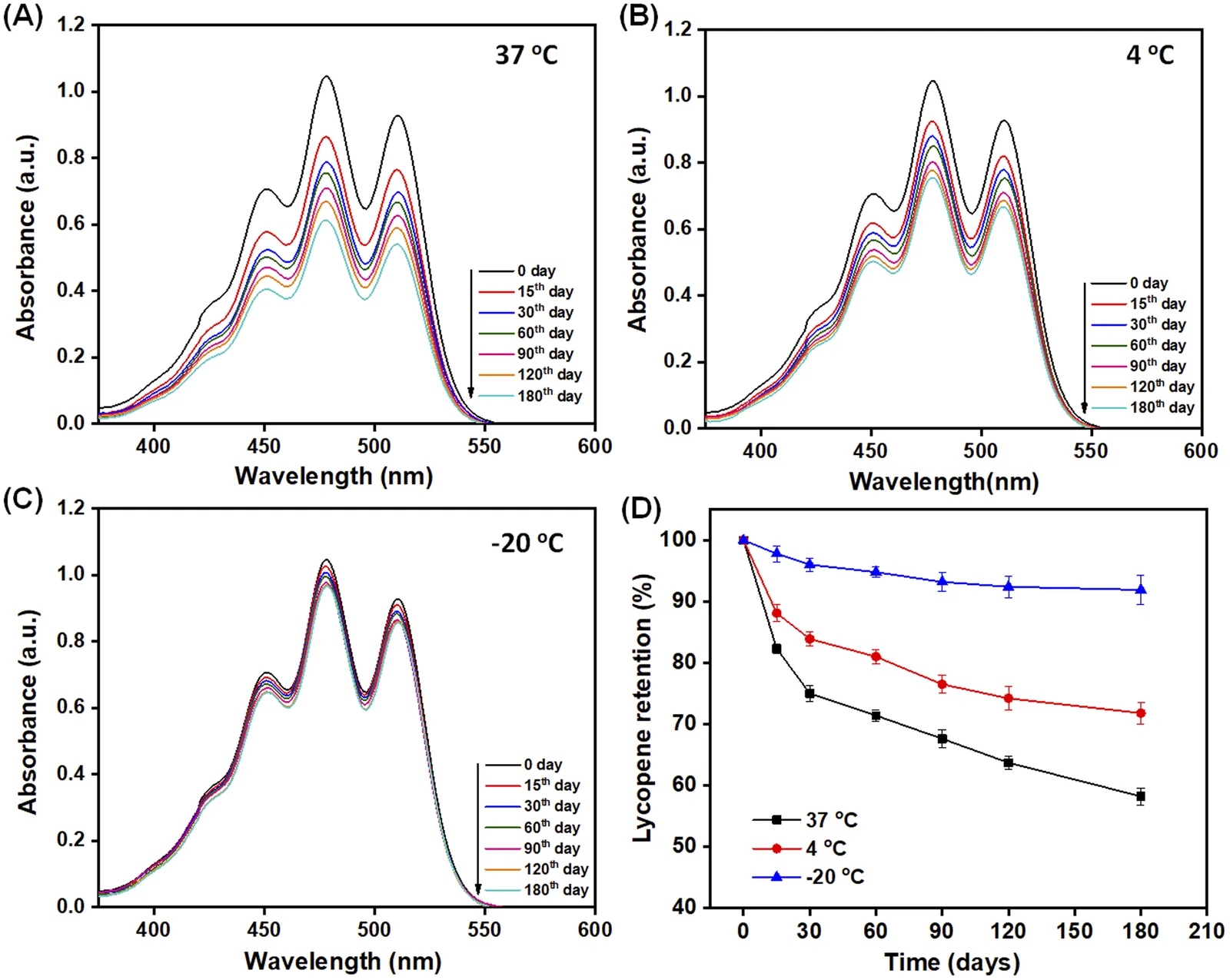
Storage stability of freeze-dried NL-5 over 180 days. UV-Vis absorption spectra recorded during storage at (A) 37 °C, (B) 4 °C, and (C) −20 °C; and (D) lycopene retention calculated from the spectrophotometrically determined lycopene content relative to the initial value. Data in (D) are presented as mean ± SD (*n* = 3).

Quantification based on the spectrophotometrically determined lycopene content showed that NL-5 retained 91.9, 71.8, and 58.2% of its initial value after 180 days at −20, 4, and 37 °C, respectively (Fig. 7D). At 4 and 37 °C, the retention curves showed a relatively rapid initial decrease followed by a slower decline, whereas loss at −20 °C was substantially smaller over the same period. The temperature dependence of lycopene retention is compatible with the known susceptibility of its extended conjugated polyene structure to degradation under thermal and oxidative stress. Previous studies of lycopene-loaded lipid nanocarriers have similarly reported greater preservation at lower storage temperatures,^27,35^ while lycopene–cyclodextrin association has been reported to improve resistance to thermal and photochemical stress.^21^

The colloidal and dry-state stability experiments therefore provide complementary information. Redispersed NL-5 maintained its colloidal characteristics more effectively at 4 °C than at 37 °C over 7 days, whereas spectrophotometrically determined lycopene retention in the freeze-dried formulation was greatest at −20 °C over 180 days. These results support refrigerated conditions for short-term storage after redispersion and frozen storage for prolonged preservation of the dry formulation.

### 3.6. Radical-scavenging activity and protection against H_2_O_2_-induced oxidative injury

The apparent radical-scavenging activity of NL-5 was evaluated using the DPPH assay and compared with that of free lycopene at matched lycopene concentrations (Fig. 8A). Both samples showed concentration-dependent activity, but NL-5 consistently produced greater DPPH scavenging across the tested concentration range. The blank formulation showed no significant activity at the corresponding excipient-equivalent concentrations, indicating that the measured response was associated primarily with lycopene rather than direct radical-scavenging activity of the carrier matrix.

**Fig. 8:**
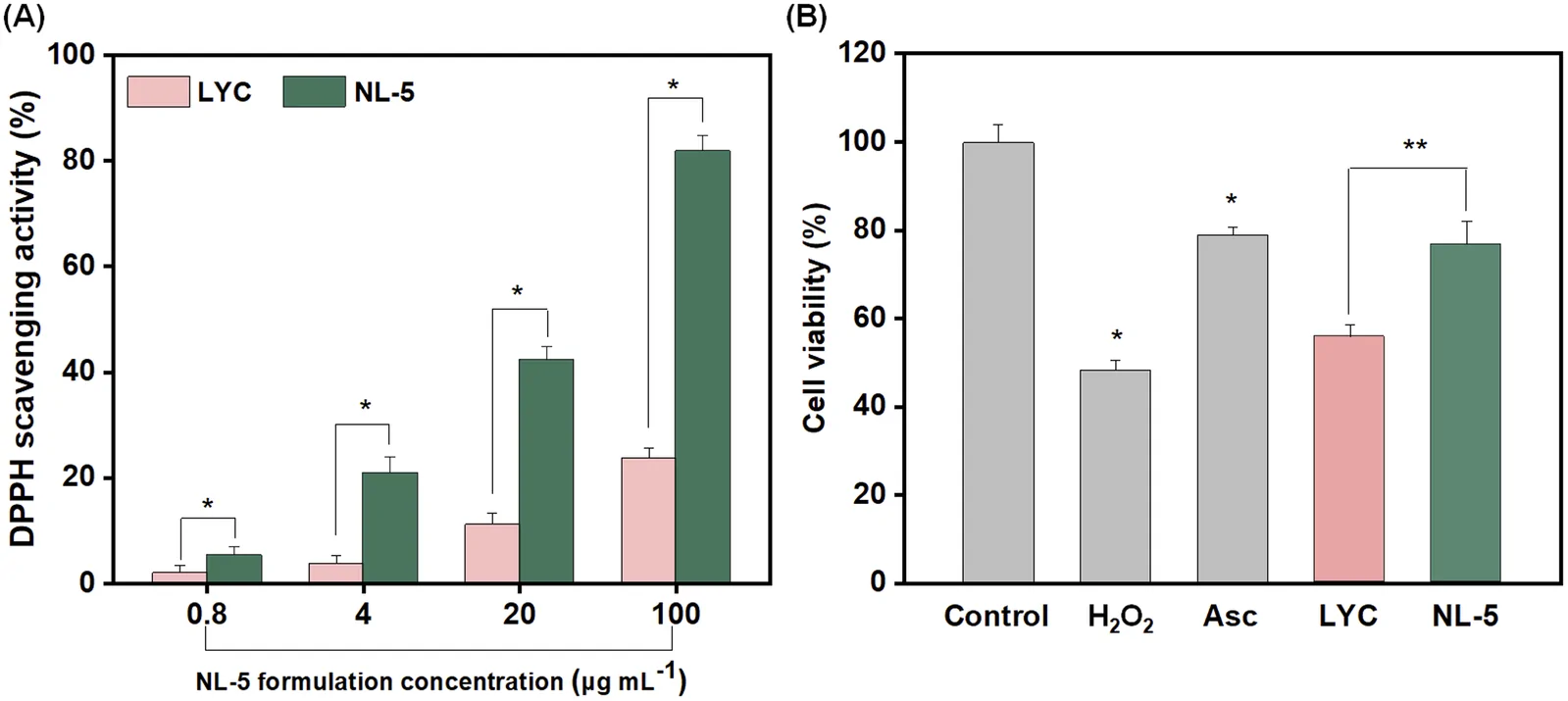
Antioxidant and cytoprotective activities of the selected lycopene nanoparticle formulation. (A) DPPH radical-scavenging activities of free lycopene and NL-5. (B) Viability of H_2_O_2_-stressed HepG2 cells following pretreatment with free lycopene and NL-5. Data are presented as mean ± SD (*n* = 3).

The biological relevance of this formulation-dependent difference was further examined in H_2_O_2_-stressed HepG2 cells (Fig. 8B). Exposure to 0.5 mM H_2_O_2_ reduced cell viability to 48.6% of the untreated control (Fig. S3). Pretreatment with free lycopene increased viability to 55.8%, whereas NL-5 increased viability to 76.8% at the same lycopene dose; ascorbic acid yielded a corresponding value of 79.2%. Thus, under the conditions of this assay, NL-5 provided greater protection against H_2_O_2_-induced loss of cell viability than free lycopene at an equivalent lycopene concentration. Previous studies have similarly reported protective effects of free or nanoformulated lycopene in H_2_O_2_-stressed hepatic and other cell models.^33,36,37^ Differences in cell type, formulation, dose, and experimental design preclude direct quantitative comparison, but these studies provide supporting context for the enhanced cellular response observed with NL-5.

### 3.7. Inhibition of intracellular lipid accumulation in HepG2 cells

To determine whether NL-5 attenuated intracellular lipid accumulation, OA-treated HepG2 cells were analyzed by Oil Red O staining (Fig. 9). NL-5 was applied at formulation concentrations of 0.8–100 µg mL^−1^, while free lycopene was included at matched lycopene concentrations calculated from the measured lycopene content of NL-5 (11.63 wt%). Both free lycopene and NL-5 reduced OA-induced intracellular lipid accumulation in a concentration-dependent manner, with consistently greater inhibition observed for NL-5 at equivalent lycopene doses (Fig. 9A). Across the tested concentration range, inhibition increased from 19.7 to 89.1% for NL-5, compared with 14.2 to 68.0% for free lycopene. These results indicate that nanoformulated lycopene produced greater attenuation of OA-induced lipid accumulation than free lycopene under the matched-dose conditions used in this assay.

**Fig. 9:**
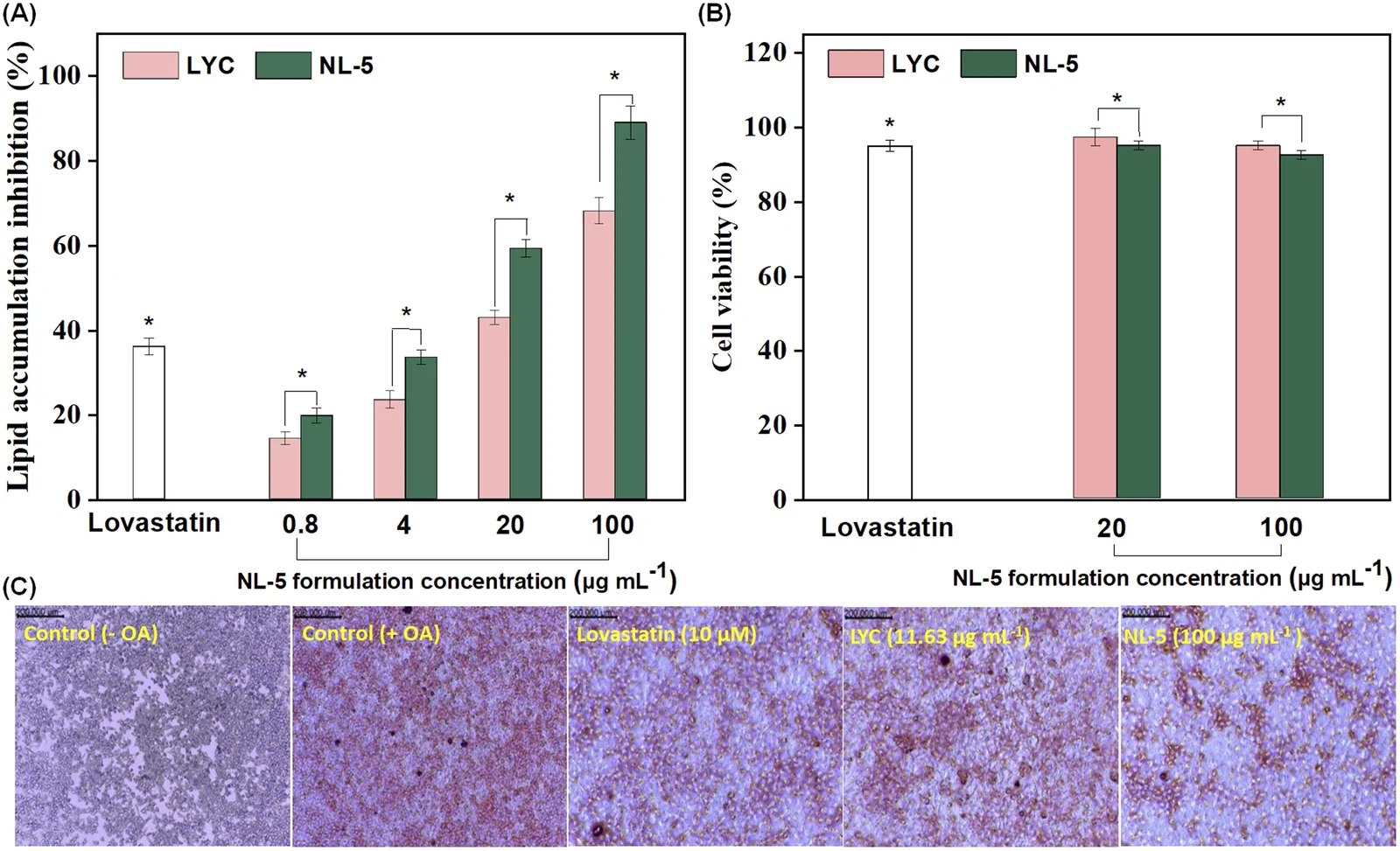
Effects of free lycopene and NL-5 on intracellular lipid accumulation in OA-treated HepG2 cells. (A) Inhibition of intracellular lipid accumulation by free lycopene and NL-5. (B) Cell viability following treatment with free lycopene and NL-5. Lovastatin (10 µM) was used as the positive control. (C) Representative Oil Red O-stained micrographs of untreated control, OA control, lovastatin-treated, free-lycopene-treated, and NL-5-treated cells. Data are presented as mean ± SD (*n* = 3).

Cell viability remained above 90% following treatment with either free lycopene or NL-5 (Fig. 9B), indicating that the decrease in Oil Red O accumulation was not primarily attributable to loss of viable cells. The blank formulation had no significant effect on either intracellular lipid accumulation or cell viability at the corresponding excipient-equivalent concentrations, supporting an association of the observed response with incorporated lycopene rather than with the carrier matrix alone. Representative Oil Red O-stained micrographs were consistent with the quantitative results, showing abundant intracellular lipid droplets in OA-treated cells and visibly reduced staining after treatment with lovastatin, free lycopene, or NL-5 (Fig. 9C). The inhibitory response observed here is consistent with previous reports that lycopene can attenuate fatty-acid-induced lipid accumulation in hepatic cell models.^38,39^ The greater response observed for NL-5 at matched lycopene doses may reflect formulation-dependent differences in how lycopene was dispersed and presented to cells under the assay conditions. However, because intracellular lycopene was not quantified, this difference cannot be attributed specifically to enhanced cellular uptake or greater intracellular lycopene exposure.

## 4 Conclusions

Redispersible lycopene nanoparticles were developed through sequential control of interfacial composition and lycopene loading. The mixed Tween 80/PC interface provided more favorable colloidal properties than either stabilizer alone, while increasing lycopene loading revealed a clear trade-off between active content and particle-size control. Within the investigated range, NL-5 provided the best balance, combining 11.63 wt% measured lycopene with low dispersity, effective redispersion, and favorable storage behavior. Structural analyses further indicated substantial suppression of detectable lycopene crystallinity within the freeze-dried matrix. At matched lycopene doses, NL-5 consistently outperformed free lycopene in DPPH radical scavenging, protection against H_2_O_2_-induced loss of HepG2 cell viability, and attenuation of OA-induced intracellular lipid accumulation. These results establish a clear formulation–property–performance relationship for the HPβCD-assisted platform and support further evaluation of NL-5 in more physiologically relevant models.

## Author contributions

Conceptualization, Mai Ha Hoang, Ho Thi Oanh; formal analysis, Ho Thi Oanh, Hong Tham Nguyen; methodology, Ho Thi Oanh, Mai Ha Hoang; project administration, Ho Thi Oanh; validation, Nguyen Phu Duc, Nhung Hac Thi, Dinh Dung Luong; writing – original draft, Ho Thi Oanh; writing – review and editing, Mai Ha Hoang, Ho Thi Oanh. All authors have read and approved the final version of the manuscript.

## Conflicts of interest

There are no conflicts to declare.

## Supplementary Material

RA-OLF-D6RA08147A-s001

## Data Availability

The data supporting this article have been included as part of the supplementary information (SI). Supplementary information is available. See DOI: https://doi.org/10.1039/d6ra08147a.
