# Supplementary material for: Formulation-dependent colloidal properties of redispersible lycopene nanoparticles: storage stability and enhanced in vitro bioactivity
Source: RSC Adv. 2026 Oct 19. Online ahead of print. doi: 10.1039/d6ra08147a (PMC13678499; doi:10.1039/d6ra08147a)
Supplement: Supplementary file 1 [file RA-OLF-D6RA08147A-s001.pdf]

Supplementary Information (SI) for RSC Advances.
This journal is © The Royal Society of Chemistry

Electronic Supplementary Information (ESI)

Formulation-dependent colloidal properties of redispersible lycopene nanoparticles: storage stability and enhanced *in vitro* bioactivity

Ho Thi Oanh,[a] Hong Tham Nguyen,[a,b] Nguyen Phu Duc,[a] Nhung Hac Thi,[a] Dinh Dung Luong,[a] Mai Ha Hoang,*[a,b]

[a]*Institute of Chemistry, Vietnam Academy of Science and Technology, 18 Hoang Quoc Viet, Nghia Do Ward, Hanoi, 10000, Vietnam.*
[b]*Graduate University of Science and Technology, Vietnam Academy of Science and Technology, 18 Hoang Quoc Viet, Nghia Do Ward, Hanoi, 10000, Vietnam.*

** Corresponding author. E-mail: hoangmaiha@ich.vast.vn*

Table of Contents

Supplementary Note S1. Spectroscopic characterization of lycopene extracted from gac aril

Fig. S1. Spectroscopic characterization of lycopene extracted from gac aril

Fig. S2. Colloidal stability of redispersed NL-5 during storage at 4 and 37 °C

Fig. S3. Concentration-dependent effect of H₂O₂ on HepG2 cell viability

Supplementary Note S1. Spectroscopic characterization of lycopene extracted from gac aril

UV–vis analysis of the isolated product showed the characteristic three-band absorption profile of lycopene and closely matched that of the authentic lycopene standard (Fig. S1A). Quantification against the standard indicated an estimated purity of approximately 98%. Extraction of 100 g of air-dried gac aril yielded 0.35 g of a product containing approximately 98% lycopene (Fig. S1A, inset).

The chemical identity of the isolated product was further confirmed by ^1H and ^{13}C NMR spectroscopy. The ^1H NMR spectrum showed characteristic olefinic resonances at δ 6.66–6.17 and 5.95 ppm, together with an additional olefinic signal at δ 5.11 ppm. The signal at δ 2.12 ppm was assigned to allylic methylene protons, whereas the resonances at δ 1.97, 1.82, 1.69, and 1.61 ppm were attributed to methyl groups along the conjugated hydrocarbon chain. The integrated resonances accounted for 56 protons, consistent with the molecular formula $C_{40}H_{56}$.

The ^{13}C NMR spectrum displayed 20 distinct resonances, including 13 olefinic signals between δ 123.98 and 139.47 ppm and seven aliphatic signals between δ 12.79 and 40.24 ppm. The 20 chemically distinct carbon environments are consistent with the high molecular symmetry of lycopene. The residual $CDCl_3$ solvent signal at approximately δ 77 ppm was excluded from the assignment. Collectively, the UV–vis and NMR data confirmed the identity of the isolated product as lycopene.

^1H NMR (600 MHz, $CDCl_3$) δ 6.66–6.17 (m, 14H), 5.95 (d, J = 11.0 Hz, 2H), 5.11 (br s, 2H), 2.12 (br s, 8H), 1.97 (s, 12H), 1.82 (s, 6H), 1.69 (s, 6H), and 1.61 (s, 6H).

^{13}C NMR (151 MHz, $CDCl_3$) δ 139.47, 137.37, 136.55, 136.16, 135.42, 132.65, 131.73, 131.56, 130.09, 125.76, 125.16, 124.81, 123.98, 40.24, 26.71, 25.69, 17.69, 16.96, 12.90, and 12.79.

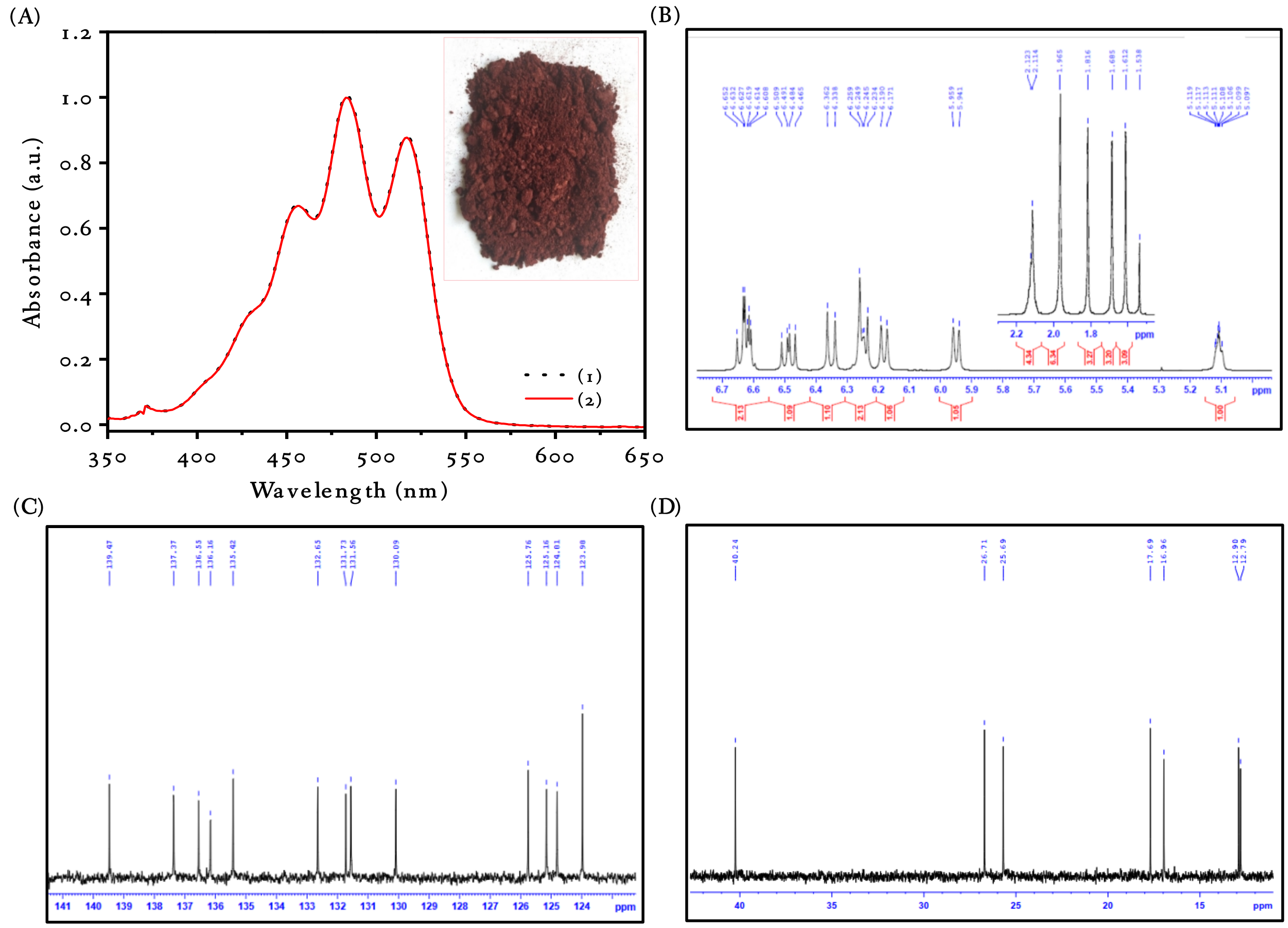

Fig. S1 Spectroscopic characterization of lycopene extracted from gac aril. (A) UV–vis spectra of a commercial lycopene standard (Sigma-Aldrich, 98% purity; 1) and the isolated lycopene (2); the inset shows the appearance of the isolated product. (B) ^{1}H NMR spectrum of the isolated lycopene recorded at 600 MHz in $CDCl_3$, with an inset showing the expanded aliphatic region. (C, D) Expanded olefinic and aliphatic regions, respectively, of the ^{13}C NMR spectrum recorded at 151 MHz in $CDCl_3$.

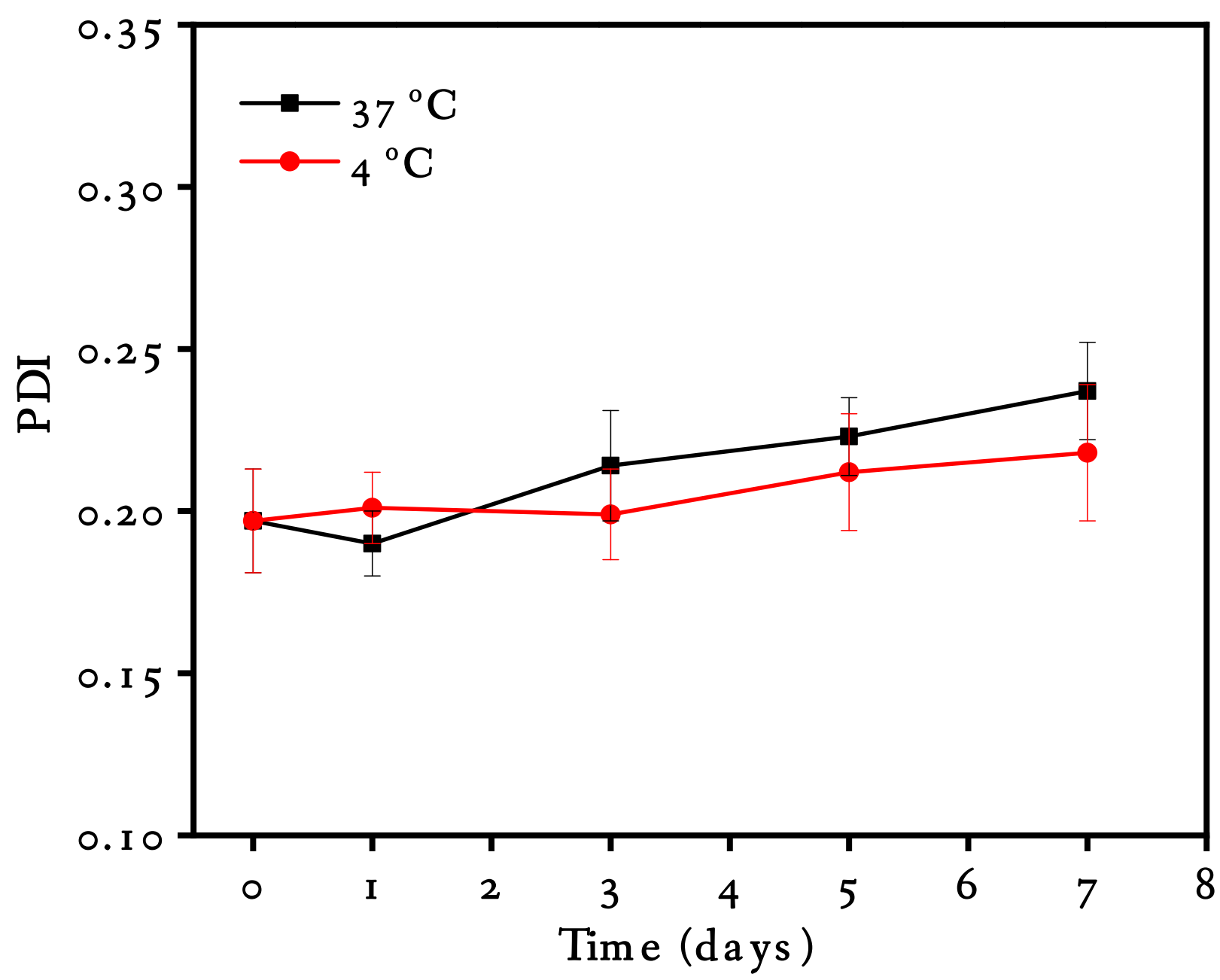

Fig. S2 Colloidal stability of redispersed NL-5 during storage at 4 and 37 °C for 7 days, expressed as changes in the polydispersity index (PDI). Data are presented as mean ± SD (n = 3).

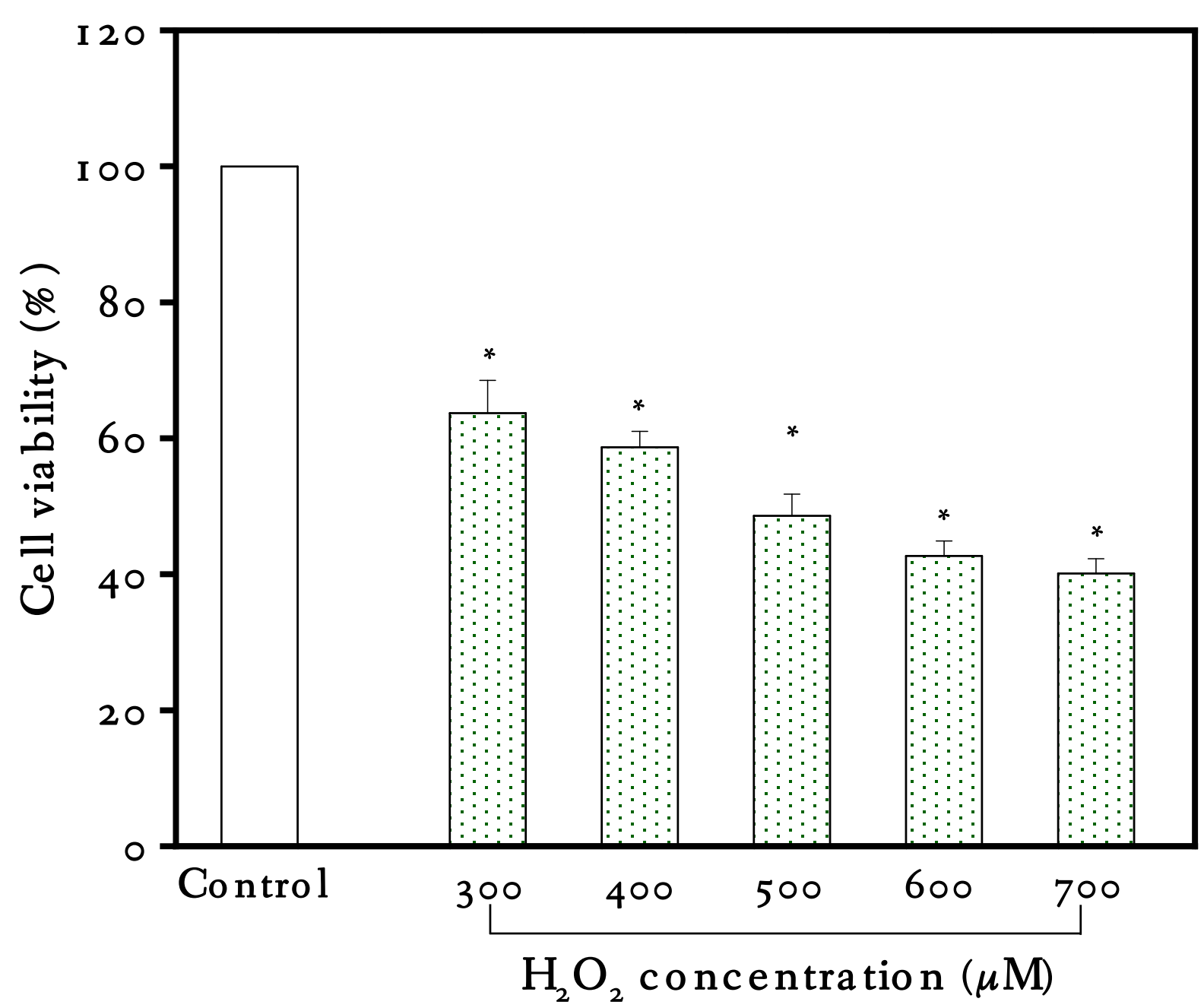

Fig.S3 Concentration-dependent effect of H_2O_2 on HepG2 cell viability. Cells were exposed to 300–700 μM H_2O_2 for 1 h. Data are presented as mean ± SD (n = 3). Based on this concentration–response experiment, 500 μM H_2O_2 was selected for the subsequent oxidative-injury experiments.
